# Experimental and Numerical Investigation of the Tensile and Failure Response of Multiple-Hole-Fiber-Reinforced Magnesium Alloy Laminates under Various Temperature Environments

**DOI:** 10.3390/ma16165573

**Published:** 2023-08-10

**Authors:** Zhongzhao Lin, Dongfa Sheng, Yuting Fang, Ke Xiong, Yuming Song

**Affiliations:** 1 School of Civil Engineering, Southwest Forestry University, Kunming 650224, Chinafangyuting@swfu.edu.cn (Y.F.); 2 National Supercomputing Center in Guangzhou, Sun Yat-Sen University, Guangzhou 510006, China; ke.xiong@nscc-gz.cn; 3 School of Chemistry and Chemical Engineering, Kunming University, Kunming 650214, China; songyumin875@126.com

**Keywords:** fiber metal laminates, multiple holes, temperature effect, tensile response, numerical simulation, damage evolution

## Abstract

In this paper, the tensile mechanical behavior and progressive damage morphology of glass-fiber-reinforced magnesium alloy laminate for different numbers of holes in a temperature range of 25–180 °C were investigated. In addition, based on extensive tensile tests, the tensile mechanical behavior and microscopic damage morphology of porous-glass-fiber-reinforced magnesium alloy laminates at different temperatures were observed by finite element simulation and scanning electron microscopy (SEM). Finally, the numerical simulation and experimental results were in good accordance with the prediction of mechanical properties and fracture damage patterns of the laminates, the average difference between the residual strength values of the specimens at ambient temperature was 5.57%, and the stress–strain curves were in good agreement. The experimental and finite element analysis results showed that the damaged area of the bonded layer tended to expand with the increase in the number of holes, which has a lesser effect on the ultimate tensile strength. As the temperature increased, the specimens changed from obvious fiber breakage (pull-out) and the resin matrix damage mode to matrix softening damage and interfacial delamination fracture damage. As the testing temperature of the specimens increased from 25 °C to 180 °C, the tensile strength of the specimens decreased by an average of 51.59%, while the tensile strength of the specimens showed a nonlinear decreasing trend. The damage mechanism of porous-glass-fiber-reinforced magnesium alloy laminates at different temperatures is discussed in this paper, which can provide a reference for engineering applications and design.

## 1. Introduction

Fiber metal laminates (FMLs) are a typical composite material consisting of alternating layers of surface-treated alloy laminate and fiber/resin material [1,2]. Based on the hybrid structure, FMLs have outstanding properties of high strength and stiffness, excellent plasticity, fatigue resistance, and corrosion resistance [3,4]. With the development of FML applications of lightweight composite materials, engineering requirements have placed higher demands on the load-bearing capacity, efficiency, and reliability of structures [5,6]. These advantages have made FMLs a potential lightweight and high-strength material for aerospace and transportation applications. However, the mechanical properties of fiber metal laminates (FMLs) are sensitive to the working environmental conditions, especially at higher temperatures where the resin matrix softens, affecting the interlaminar bond strength and transverse tensile strength [7,8,9]. Therefore, it is necessary to further investigate the tensile behavior and damage mechanism of glass-fiber-reinforced magnesium alloy laminates at different temperatures and numbers of holes to promote the application of FMLs in engineering.

With the rising international oil prices, reducing the structural weight of vehicles can effectively reduce fuel consumption, so there is a great demand for lightweight and high-strength equipment in aerospace, transportation, and other fields, and FML has great application value due to its lightweight and high-strength mechanical properties. Cortes et al. [10] proposed a research article on the tensile strength and impact energy absorption properties of fiber-reinforced magnesium composite laminates, and the results showed that increasing the volume percentage of the FML fiber layer would significantly enhance its tensile strength, while the fiber layer could significantly enhance the fatigue resistance. In addition, the delamination and shear fracture phenomena presented in the magnesium alloy layer could significantly enhance the impact energy absorption capacity of the laminate. The study of Cao et al. [11] experimentally investigated the residual tensile strength of CFRP, GFRP, and hybrid fiber materials at 16–160 °C. The results showed that it significantly decreased in the composites with the increase in temperature, and remained constant when the glass transition temperature (*T_g_*) of the resin was reached. Rans et al. [12] developed a crack extension prediction model for metal fiber laminates that accurately predicts the crack growth direction and the rate of delamination extension between plies, as well as the cured residual stresses within the panels in different-temperature environments, but the prediction model was conservative in low-temperature environments. Wang et al. [13] investigated the damage modes of FMLs under the influence of geometric notched conditions and showed that the response of tensile strength to off-axis and tilt angles was greater than that to the length perimeter ratio, and as the off-axis angle increased, the laminate failure mode components developed from fiber-ply-driven to aluminum-alloy-ply-driven, and the failure modes changed from tensile to tensile–shear failures. Jakubczak [14] demonstrated through 1500 thermal cycling experiments on glass-laminate-modified aluminum carbon laminates that the number of thermal cycling has a small effect on the FML interlaminar shear strength, and the difference in ILSS strength is in the range of 10% (min. ILSS 317 MPa, max. 356 MPa).

**Figure 1:**
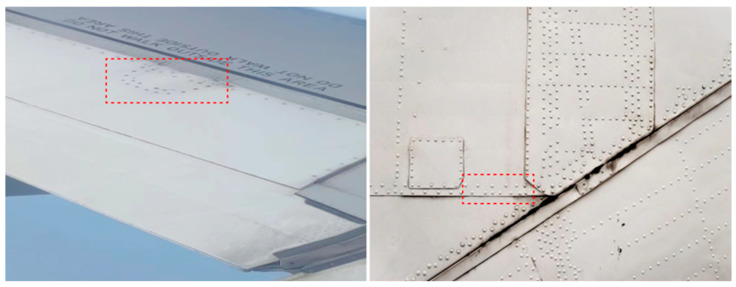
Porous structures on airplane fuselage (red box: aircraft rivet structures).

Porous structures are more common in engineering applications. The study of the damage modes and residual strength of porous structures is now supported by published literature. Gerendt et al. [15] presented a study about a finite element simulation model to predict the progressive damage failure mode of bolted connections in fiber metal laminates, and the numerical simulation and experiments were in good agreement, which can accurately predict the failure behavior of bolted connections of FMLs. Jiang et al. [16] experimentally and numerically studied the tensile mechanical behavior and progressive damage modes of Glare. Their result showed that the damage pattern of both square and round holes is determined by the “butterfly” to “funnel” pattern and square holes can lead to more fiber damage. Kazemahvazi et al. [17] investigated the influence of the layout as well as the number of holes on the residual tensile strength of composite laminates and established a damage residual strength analysis model that can effectively predict the mechanical properties of the laminates. At present, the above studies mainly focus on single-pore FMLs, and there are fewer studies on the residual mechanical properties and damage failure mechanisms of porous FMLs. Nevertheless, current research on the tensile residual strength of porous FMLs has focused on the room-temperature environment. There are also fewer studies on the mechanical properties and residual tensile strength of porous structures at different temperatures. Since porous structures are more widely used in practical engineering applications, it is essential to explore the tensile mechanical response and progressive failure mechanism modes of porous FMLs. The schematic diagram of porous structures on aircraft is shown in Figure 1.

FMLs consist of metal layers, fiber composites, and resin matrices. Previous research has shown that both the resin matrix material and fiber material are sensitive to different-temperature environments [11,18]. Fibers, metals, and resin substrates typically have different coefficients of thermal expansion. Additionally, the interfacial layer inside FMLs experiences additional thermal stresses, resulting in changes to the mechanical properties of the laminate when the working environment temperature fluctuates [19]. Ahmed et al. [20] conducted a review of the tensile mechanical behavior of FRP material under the influence of different strain rates, temperatures, and coupling effects. Their research identified failure modes of FRP materials under various strain rates and temperature conditions, and fiber cracking and fiber fracture are the main mechanisms of material failure at different strain rates, respectively. Sun et al. [21] utilized techniques such as Dynamic Mechanical Analysis (DMA), Scanning Electron Microscopy (SEM), and Digital Imaging (DIC) to investigate the effect of the environment from a low temperature of −30 °C to a high temperature of 160 °C on the tensile mechanical behavior of CFRP, and the results showed that its tensile strength and elastic modulus were reduced by 39.2% and 40% at 130 °C, respectively, and the tensile strength exhibited a nonlinear decrease with the increase in temperature. Numerous studies have shown that, especially at higher temperatures, the load-carrying capacity and failure mechanism of FMLs can be greatly affected by the different coefficients of thermal expansion of metals and epoxy resins, leading to the generation of microcracks, and thus this factor plays a key role in the mechanical characterization of FMLs at higher temperatures [22,23,24,25]. At present, some studies have shown that the density tensile strength ratio and impact absorption capacity of magnesium-based-fiber-reinforced metal laminates are stronger than those of aluminum-based fiber metal laminates [10,26,27]. Therefore, this work investigated the tensile residual properties of magnesium-based-fiber-reinforced metal laminates, which can have a positive effect on promoting the diverse applications of FML.

In this work, the tensile mechanical response and failure modes of glass-fiber-reinforced magnesium alloy laminates under different-temperature environments were investigated mainly through experimental characterization and numerical simulation. Among them, the FML specimens after tensile damage were characterized by the SEM technique for microscopic damage modes, including fiber fracture, matrix damage, fiber pull-out, and interlayer delamination. A finite element model was also developed to predict the mechanical response and progressive damage failure modes of FMLs in an ambient environment, and the finite element model considered the progressive failure evolution modes of alloy and composite layers. The above study filled the gap in understanding the failure mechanism of glass fiber magnesium alloy laminates under different-temperature working environments, and the established finite element simulation model could better predict its mechanical properties and damage modes, which is very necessary for the design and operation of FML structures under different operating environments. In addition, such a study can also help to promote the expansion of FMLs in engineering applications.

## 2. Sample Preparation and Experimental Description

### 2.1. FML Specimen Preparation

In this study, glass fibers and epoxy resin were prepared in advance shots. The composite laminate consisted of glass/epoxy resin, which was fully impregnated with epoxy resin and hardener and preheated according to a preset process. The glass fiber prepreg was from Nanjing Xinhe (Nanjing, China), and the orientation of the glass fiber epoxy prepreg used in the experiments was [90°/0°]. FML specimens were prepared by hand lay-up in the order of [Mg/90°/0°/Mg/0°/90°/Mg]. The layer thickness of glass/epoxy was 0.16 mm, and the mechanical property parameters of GFRP layers are shown in Table 1.

The magnesium alloy was AZ31B with a thickness of 0.5 mm. And the mechanical property parameters of the AZ31B magnesium alloy sheet are shown in Table 2.

**Figure 2:**
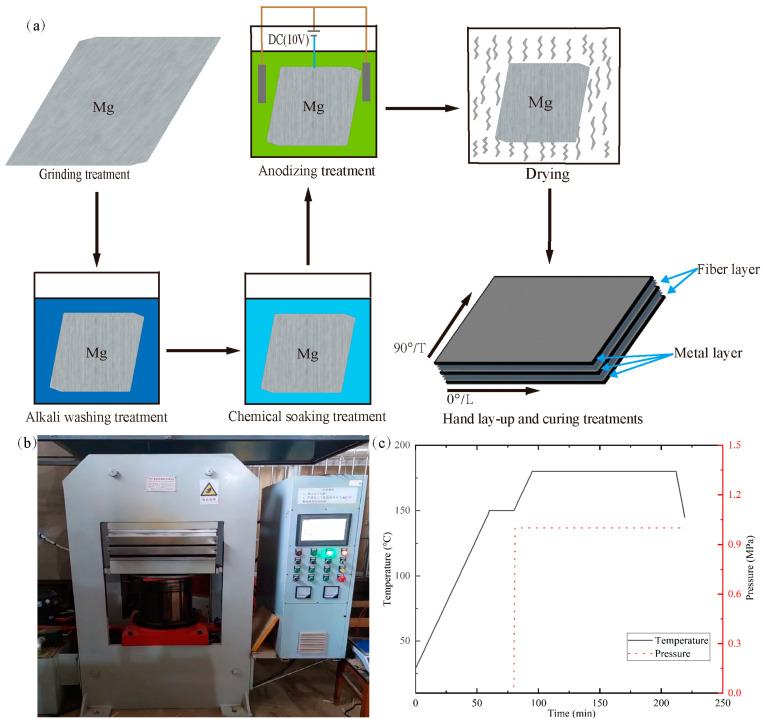
The preparation process of FML specimens. (**a**) FML preparation process, (**b**) curing machine, (**c**) curing processes.

The tensile mechanical properties of glass-fiber-reinforced magnesium alloy laminates are affected by the interlayer bonding problem between the GFRP layer and the magnesium layer. In order to improve the bonding between the fiber/epoxy layer and the magnesium alloy layer, we improved the preparation process of fiber-metal laminates to reduce the delamination phenomenon. First, the surface of the magnesium alloy layer in contact with the fiber layer was sandblasted and polished. Then, the surfaces of the magnesium alloy layer and the fiber layer were cleaned and dried. Next, the surface of the magnesium alloy plate was soaked with a chemical solution, the surface was cleaned with acetone, and after cleaning, it was put into the DHG-9030A blow drying oven for drying. Finally, the surface of the magnesium alloy layer and fiber prepreg was uniformly coated with a layer of interfacial resin, and then the surface of the magnesium alloy layer and fiber prepreg was spread manually according to the spreading order (Mg/90°/0°/Mg/0°/90°/Mg) and put into a hot molding press for curing according to the parameters of Figure 2c. Details of the preparation process and surface treatments are shown in Figure 2a below.

Figure 2b,c illustrate the detailed information of the FML thermoforming curing device and parameters. At the beginning of the rise in temperature of the equipment, the hot molding layer was subject to preheating, and the pressurized thermal curing process was carried out when it was time to put a good layer of specimens into the test piece.

**Figure 3:**
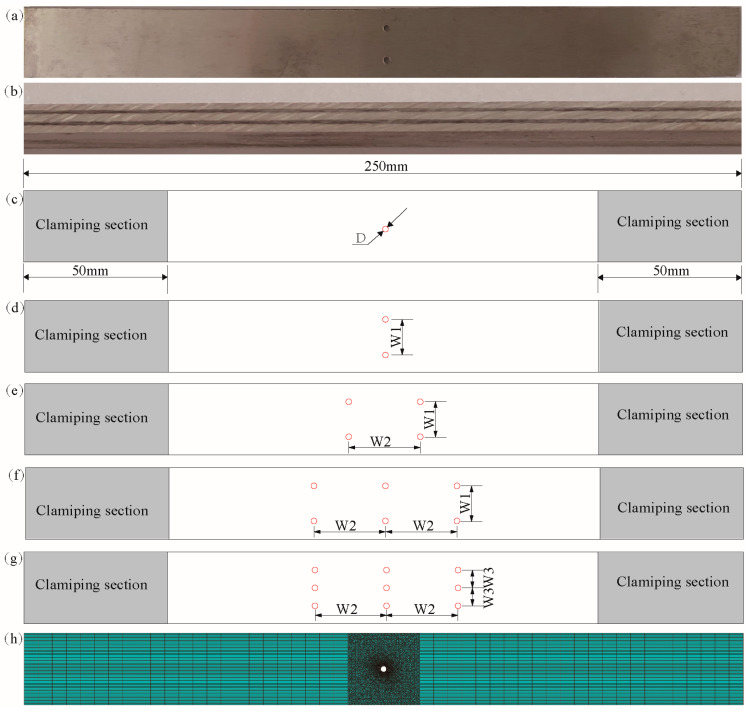
Detailed information of FML specimens. (**a**) Tensile specimen, (**b**) side of the specimen, (**c**–**g**) specimen size, (**h**) numerical analysis model of the specimen (D: diameter).

FMLs with different spacing and numbers of holes were fabricated and used to study the tensile mechanical behavior and damage failure mechanisms, as shown in Figure 3c–g. In this study, a metal CNC machine (Nanjing, China) was used to cut and punch out suitable specimens from the prepared standard FML specimens, and the preparation of the round holes was also realized by CNC machines. The dimensional parameters of the FML specimens complied with the ASTM D3039 standard [28], where the specimens were 250 mm in length and 25 mm in width. The clamping length was 25 mm and the hole spacings were w1 = 12.5 mm, w2 = 25 mm, and w3 = 6.25 mm. Finally, the specimens were subjected to surface burr polishing for removal.

### 2.2. Test Method

**Figure 4:**
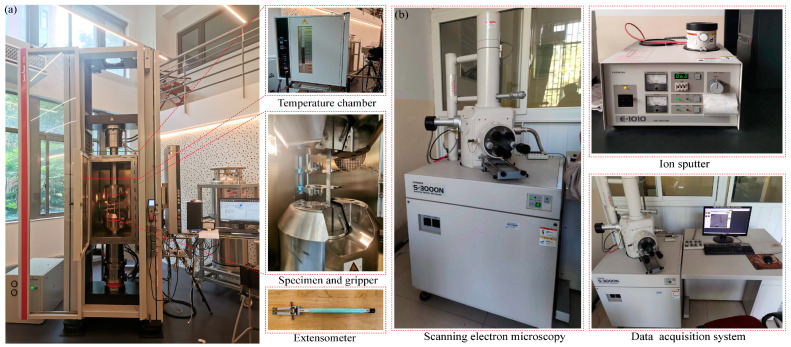
Test methods of FML specimens. (**a**) Zwick/Roell tensile test machine and FML specimen, (**b**) SEM test of fracture specimens.

In this paper, the quasi-static tensile performance experiments were performed by the Zwick/Roell Z250 tensile testing machine (Ulm, Germany) at a loading rate of 2 mm/min. The Zwick/Roell tensile test machine for tensile testing is presented in Figure 4a, and the maximum load capacity of the electronic universal testing machine is 250 kN. The fixture ensures that the specimens are in the center and vertical position. The tensile electronic universal testing machine is equipped with a temperature chamber that allows experiments to be performed at a set temperature. Figure 4a shows the specimens to be tested for tensile properties in the heating chamber. The temperature variation of the specimens in this study ranged from 25 °C to 180 °C. In addition, the heating rate of the temperature chamber was set to 5 °C/min. This temperature range provided good coverage of the potential operating ambient temperature of FMLs and enabled the exploration of damage conditions and mechanical property changes at higher temperatures.

To further analyze the failure modes of the FML specimens, small specimens of 5 mm in length and 5 mm in width were cut from the fracture of the specimens that had been subjected to tensile testing and fixed on the specimen stage with carbon conductive tape. Then, gold was sprayed on the test specimens using an ion-sputtering apparatus to improve the visibility of the SEM. Finally, the specimen was put into the machine and operated for observation according to the SEM standard procedure. The SEM equipment in this work is shown in Figure 4b.

## 3. Finite Element Numerical Simulation Model and Damage Failure Criterion 

The FEM software Abaqus/Explicit 2021 was used to investigate the progressive damage modes and failure behavior of the FMLs, which consisted of a fiber layer, a magnesium alloy layer, a resin layer, and an adhesive layer. The progressive damage modes of these layers were then defined by integrating the following damage models into the VUMAT subroutine.

### 3.1. Fiber Layer Damage Failure Model 

In this article, the three-dimensional Hashin failure criteria [29,30] were used as failure criteria for fiber layers, and the following failure modes were mainly considered, fiber tensile failure, matrix tensile fracture, and interlayer delamination failure, as shown in the following equation.

Fiber tensile and compression criterion:

(1) $$F_{f}^{t}={\left(\frac{\sigma_{11}}{X_{T}}\right)}^{2}+{\left(\frac{\tau_{12}}{S_{12}}\right)}^{2}+{\left(\frac{\tau_{13}}{S_{13}}\right)}^{2}\geq \;1\;(\sigma_{11}\;>\;0)$$

(2) $$F_{f}^{c}={\left(\frac{\sigma_{11}}{X_{C}}\right)}^{2}\geq \;1\;(\sigma_{11}\;<\;0\;)$$

Matrix tensile and compression criterion:

(3) $$F_{m}^{t}={\left(\frac{\sigma_{22}+\sigma_{33}}{Y_{T}}\right)}^{2}+\frac{1}{S_{23}^{2}}\left(\tau_{23}^{2}-\sigma_{22}\sigma_{33}\right)+{\left(\frac{\tau_{12}}{S_{12}}\right)}^{2}+{\left(\frac{\tau_{13}}{S_{13}}\right)}^{2}\geq \;1\;(\sigma_{22}+\sigma_{33}>0)$$

(4) $$F_{m}^{c}=\frac{\sigma_{22}+\sigma_{33}}{Y_{C}}\left[{\left(\frac{Y_{C}}{2S_{23}}\right)}^{2}-1\right]+\frac{{\left(\sigma_{22}+\sigma_{33}\right)}^{2}}{4S_{23}^{2}}+\frac{\tau_{23}^{2}-\sigma_{22}\sigma_{33}}{S_{23}^{2}}+{\left(\frac{\tau_{12}}{S_{12}}\right)}^{2}+{\left(\frac{\tau_{13}}{S_{13}}\right)}^{2}\geq \;1\;(\sigma_{22}+\sigma_{33}<0)$$

Delamination failure criterion:

(5) $$F_{l}^{d}={\left(\frac{\sigma_{33}}{Z_{T}}\right)}^{2}+{\left(\frac{\tau_{13}}{S_{13}}\right)}^{2}+{\left(\frac{\tau_{23}}{S_{23}}\right)}^{2}\geq \;1\;(\sigma_{33}>0)$$

In Equations (1)–(5). $\sigma_{ij}$ and $\tau_{ij}$ are the stress in each direction of the composite laminate and the shear stress. $X_{C}$, $X_{T}$, $Y_{C}$, $Y_{T}$, $Z_{C}$, and $Z_{T}$ are the composite layers in the relevant direction of tensile and compressive strengths, respectively. $S_{\mathrm{ij}}$ is the corresponding surface shear strength.

### 3.2. Metal Layer Damage Failure Criterion

For magnesium alloy layers, the damage evolution can be implemented using the Johnson–Cook ductile damage model [31,32] in Equations (6) and (7). The magnesium alloy layer in the fiber metal laminate is defined as an elasticity and plasticity material.

(6) $$\bar{\sigma }=\left[A+B{\left({\bar{\varepsilon }}_{\mathrm{pl}}\right)}^{n}\right]\left[1+C\ln\left(\frac{{\dot{\bar{\varepsilon }}}_{\mathrm{pl}}}{{\dot{\varepsilon }}_{0}}\right)\right]$$

(7) $$\omega =\sum \frac{\Delta {\bar{\varepsilon }}^{\mathrm{pl}}}{{\bar{\varepsilon }}_{f}^{\mathrm{pl}}}\;\mathrm{with}\;{\bar{\varepsilon }}_{f}^{\mathrm{pl}}=\left[d_{1}+d_{2}\exp\left(d_{3}\sigma^{*}\right)\right]\left[1+d_{4}\ln\left(\frac{{\dot{\bar{\varepsilon }}}^{\mathrm{pl}}}{{\dot{\varepsilon }}^{0}}\right)\right]$$

where $\bar{\sigma }$ is the yield stress. ${\bar{\varepsilon }}_{\mathrm{pl}}$ and ${\dot{\bar{\varepsilon }}}_{\mathrm{pl}}$ are the equivalent plastic strain and equivalent plastic strain rate, respectively. ${\dot{\varepsilon }}_{0}$ represents the reference strain rate. *A*, *B*, and *n* are constitutive model parameters. For defining the magnesium layer in FMLs, ω is the damage accumulative factor; if ω exceeds one, the simulation program then executes the grid deletion procedure. The Johnson–Cook ductile damage model was proved an accurate description of the damage evolution of the magnesium alloy layer. Finally, *A*, *B*, *n*, *c*, *m*, and $d_{1}-d_{5}$ parameters are shown in Table 3 [33].

### 3.3. Interlaminar Delamination Model

The bonding interface failure between laminates is determined by the bilinear adhesion zone, where the elasticity of the viscous elements can be defined by the traction separation law, as follows [34]:

(8) $$\sigma =E\varepsilon =\left[\begin{array}{c} \sigma_{n}\\ \sigma_{s}\\ \sigma_{t} \end{array}\right]=\left[\begin{array}{ccc} E_{\mathrm{nn}} & 0 & 0\\ 0 & E_{\mathrm{ss}} & 0\\ 0 & 0 & E_{\mathrm{tt}} \end{array}\right]\left[\begin{array}{l} \varepsilon_{n}\\ \varepsilon_{s}\\ \varepsilon_{t} \end{array}\right]$$

where σ is the nominal stress vector and ε is the nominal strain vector. E is the elastic stiffness matrix, where $E_{\mathrm{nn}}$, $E_{\mathrm{ss}}$, and $E_{\mathrm{tt}}$ represent the penalty stiffness and $\sigma_{n}$, $\sigma_{s}$, $\sigma_{t}$ indicate the interface traction stresses in their direction, respectively.

The damage onset is determined by the following Benzeggagh–Kenane failure criterion [35]:

(9) $${\left\{\frac{\sigma_{n}}{\sigma_{n}^{0}}\right\}}^{2}+{\left\{\frac{\sigma_{s}}{\sigma_{s}^{0}}\right\}}^{2}+{\left\{\frac{\sigma_{t}}{\sigma_{t}^{0}}\right\}}^{2}=1$$

(10) $$G_{IC}+\left(G_{\mathrm{II}C}-G_{IC}\right){\left(\frac{G_{\mathrm{II}}+G_{\mathrm{III}}}{G_{I}+G_{\mathrm{II}}+G_{\mathrm{III}}}\right)}^{\eta }=G_{C}$$

where $G_{\mathrm{IC}}$, $G_{\mathrm{IIC}}$, and $G_{\mathrm{IIIC}}$ denote the dissipation energy of cohesive elements in different directions and C represents the critical fracture energy. The cohesion layer properties are listed in Table 4 [36].

### 3.4. Finite Element Numerical Simulation Model

In this work, the VUMAT subroutine was written and imported into the finite element analysis software ABAQUS 2021 for numerical simulation and analysis, the finite element solid cell model was established, and the material property parameters were assigned to the glass fiber layer, resin bonded layer, and magnesium alloy layer in the direction of plate thickness. Solid modeling of the magnesium alloy layer and fiber layer was conducted using isotropic and orthotropic anisotropic elastic models, respectively. The mesh division of the fiber layer and magnesium alloy layer was carried out using the C3D8R reduced integral cell, the bonding layer of both was carried out using the COH4D8 integral cell, and the mesh refinement was carried out at the place where stress concentration exists at the hole edge.

## 4. Analysis of Finite Element Simulation Results

As the fiber-metal laminate is a sandwich structure, the fiber layer and part of the metal layer are located inside the laminate. And the progressive damage of the fiber-metal laminate is a dynamic process, so it is difficult to observe the progressive damage extension of this part in the experiment. In contrast, the FEM can observe the progressive damage extension process of any layer. Therefore, finite element simulation analysis can effectively study the deformation and progressive damage process of the fiber layer inside the fiber-metal laminate. In this part, the FEM results of five specimens with different numbers of holes at room temperature (25 °C) were compared with the results of experiments to further explore the damage mechanism of FMLs containing holes. It is well known that the damage evolution of magnesium alloy layers and fiber layers during tensile testing is complex. Therefore, in this study, the progressive failure analysis of FMLs based on the progressive damage model of the magnesium plate, composite layer, and interlayer bond was carried out to clarify the damage mechanism and failure sequence.

### 4.1. Comparison of Finite Element Results with Experiments

In this section, to study the interlayer damage pattern of FML specimens, a chemical treatment was used to remove the metallic layers from the surface of the specimens so that the fibrous and cohesive layers could be better observed.

**Figure 5:**
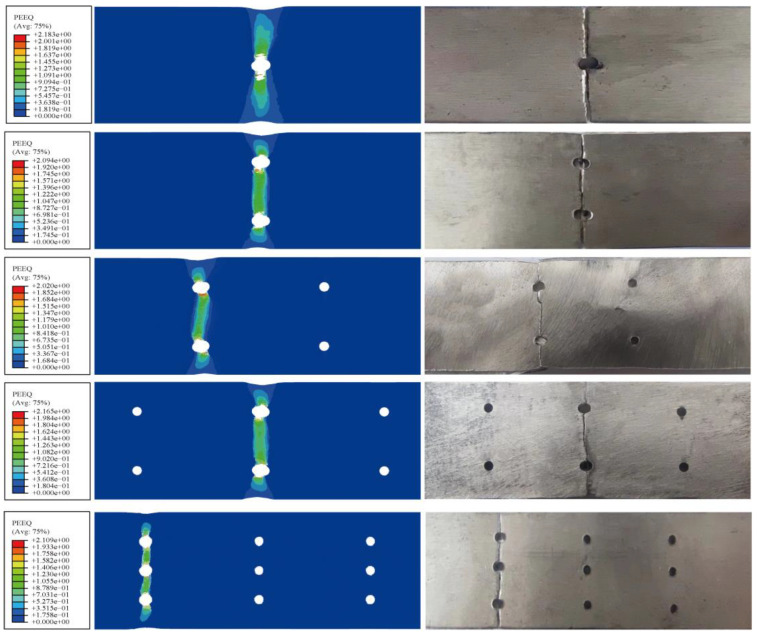
Comparison of equivalent plastic strain damage in the tension of FML specimens with holes.

According to Figure 5, showing the details of the FML specimens after failure and the comparison of the PEEQ-equivalent plastic strain field, the equivalent plastic strain region of the magnesium alloy is the same as the fracture region in the experiment. Observations reveal significant plastic deformation in the magnesium alloy layer under tensile load, and the crack in the specimen progressively propagates from the edge of the hole to the surrounding area perpendicular to the applied load. As the analysis steps and load increase, the plastic damage area of the metal layer visibly expands, there is a plastic yielding and necking phenomenon in the cross-section of the magnesium alloy layer, and the fracture mode is accompanied by a certain ductile fracture of delamination phenomenon. A comparison between the PEEQ-equivalent plastic strain diagram and the experimental diagram confirms the consistent expansion of plastic cumulative deformation in the magnesium alloy layer with the observed fracture damage range. In addition, plastic deformation of the metal layer perpendicular to the stress direction produces a stress redistribution, which reduces the stress concentration effect at the hollow edge.

**Figure 6:**
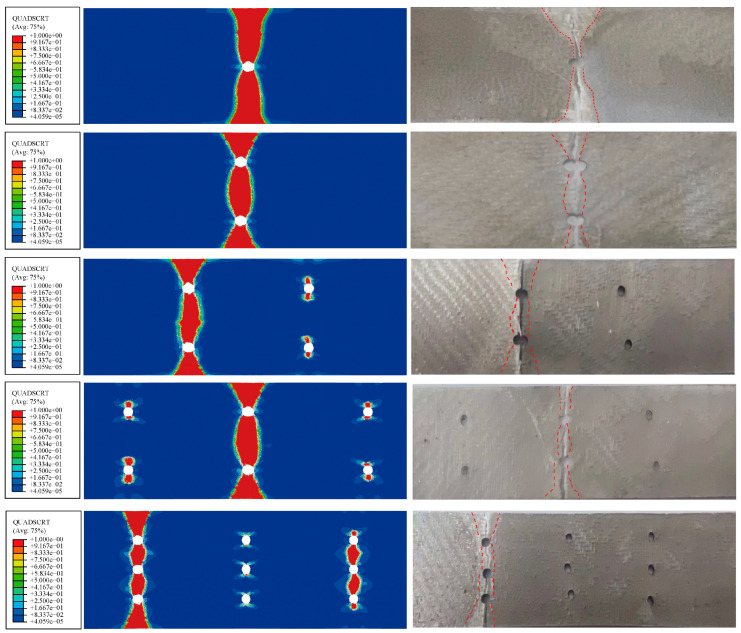
Comparison of interlayer damage between numerically simulated and experimental FML specimens (red dashed line: interlayer failure area).

The QUADSCRT diagram illustrates the failure of the bonding layer between the fiber and the two layers; when this area is fully damaged with a value of 1, it is shown in red. The interlayer failure of the FML specimens is shown in Figure 6, where the delamination damage area between the specimen layers shows a funnel shape. From the analysis, it can be concluded that with an increase in the number of holes, the premature fracture damage area of the specimens decrease. From the comparison of the numerical simulation and experimental results, it can be seen that there are delamination damage regions at the fracture edge and hole edge of the specimen. The whitened area on the experimental graph is the failure area of the adhesive layer, and the comparison between the red failure area of the numerical simulation and the white area of the experiment is more consistent, which shows that the numerical simulation can better predict the interlayer failure mode. The delamination damage zone develops gradually from the edge of the hole along 90° to the edge of the specimen as the load gradually increases, and the area between the holes has a high incidence of delamination damage. Moreover, the stiffness of porous FML specimens decreases with the increasing number of holes under identical temperature conditions.

### 4.2. Analysis of Finite Element Simulation Results

**Figure 7:**
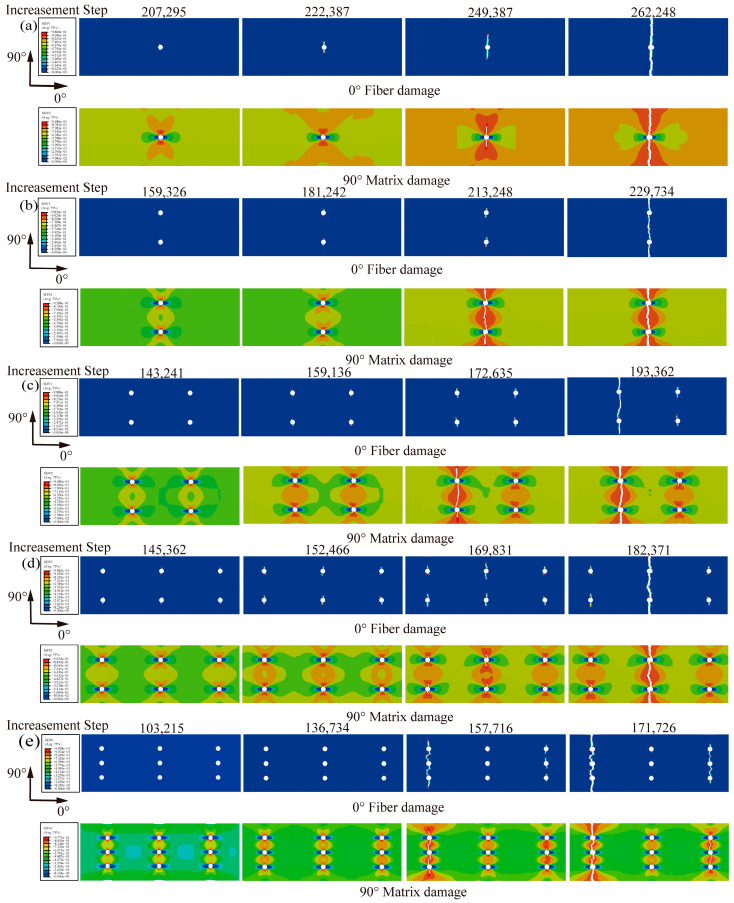
Damage evolution of composite layers, (**a**) single hole, (**b**) two holes, (**c**) four holes, (**d**) six holes, (**e**) nine holes.

SDV is the damage state parameter of the composite layer defined by the VUMAT subroutine, where SDV1 and SDV2 represent the damage state of the fiber and the damage state of the matrix, respectively. The fracture of the finite element numerical model is controlled by unit deletion; when the damage state reaches the unit failure, the unit deletion step is performed, and the unit no longer bears or transmits the load. Figure 7 shows the rainbow diagram of the damage evolution of the 0° fiber layer (SDV1) and the 90° matrix layer (SDV2) of the FMLs. Initially, the cohesive unit in the 90°/T direction of the circular hole at the edge of the hole was partially damaged, and then the crack expanded to both sides of the longitudinal direction, and the extent of damage failure gradually expanded from the “X shape” to the “funnel shape”. Subsequently, the element damage first appeared on the bonding layer of the fiber layer and the metal layer without plastic deformation or fiber (resin matrix) damage to the magnesium and fiber layers. As the tensile load on the specimen increased, the damaged units gradually expanded outward, and the matrix failure of the 90° fiber layer extended gradually from the hole edge to the funnel shape. Simultaneously, as the load continued to increase, the matrix of the 90° fiber layer gradually failed and the damaged area of the 0° fiber layer began to gradually expand. Eventually, the 0° fiber layer reached the load-carrying capacity limit and damage occurred, the composite layer fractured as a whole, while the magnesium alloy layer also exhibited plastic fracture, and the whole FML specimen failed and fractured. This behavior can be attributed to the fact that the fracture strain of the magnesium alloy is significantly greater than that of the fiber layer.

The numerical simulation of damage extension analysis is in better agreement with the experiment and better simulates the load-bearing damage pattern of FMLs.

## 5. Analysis of Experimental Results

### 5.1. Microscopic Damage Mechanism of the Fractured Part

Due to the complexity of the damage of FMLs, some microscopic aspects of the damage were not obvious, so the microscopic aspects of the failure modes can be better observed using scanning electron microscopy (SEM).

**Figure 8:**
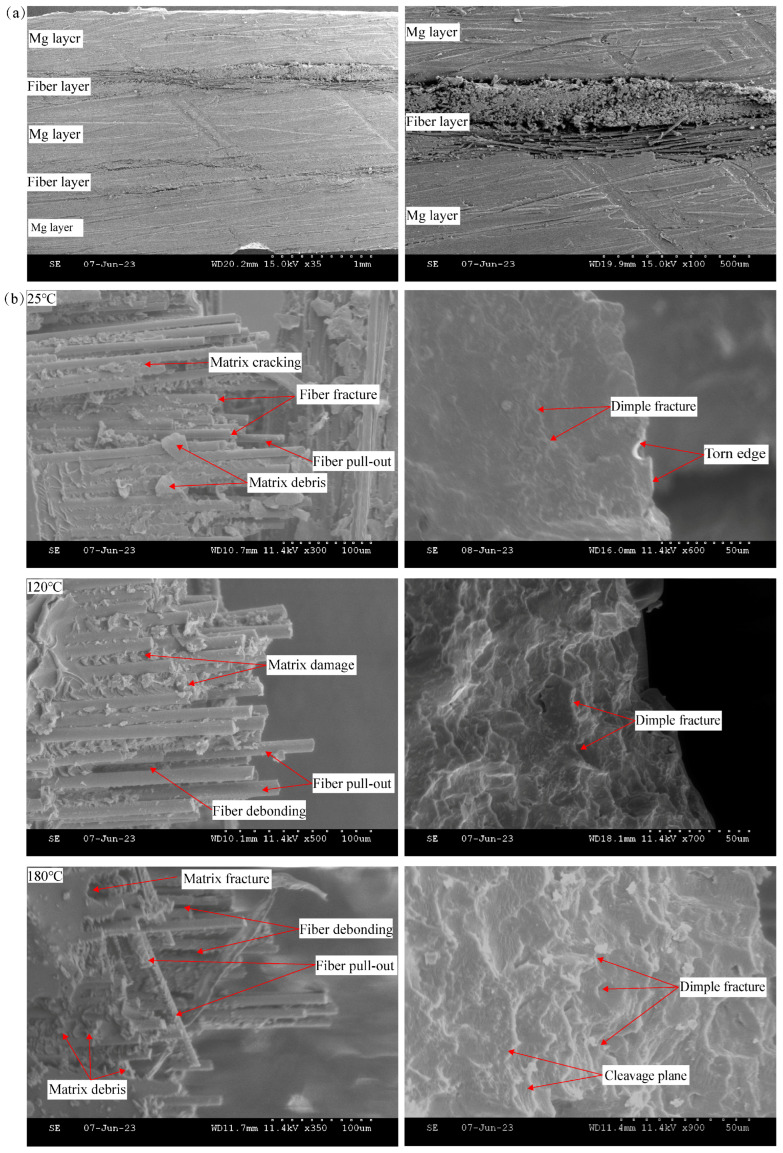
Fracture micromorphology of FML specimens. (**a**) Side microscopic graph, (**b**) microstructure diagram of fracture failure.

According to Figure 8a, the lateral microscopic morphology of the surface-treated FMLs was shown to have better interlaminar bond integrity, which effectively reduced the defects such as interlayer delamination and wrinkles and micro-pores, and improved the tightness and integrity of the interlayer of the specimen.

As shown in Figure 8b, showing the FML fracture failure microscopic morphology, it can be observed that there were smooth fiber pull-out and fractures in the fracture region of the specimen due to failure conditions such as fiber/matrix debonding. Initially, the failure of the resin matrix first resulted in fiber debonding and pulling out, showing resin matrix fragments and fiber separation around the fibers. During the damage process, there existed a stress transmission process of the resin matrix to glass fiber in the sequence of resin matrix–fiber layer–metal layer. Therefore, the tensile damage modes of FMLs were more complicated because the stress gradient between different layers was different and the fiber layer broke earlier than the metal layer. As the temperature increased and when the temperature exceeded 120 °C, it can be seen that the fiber fracture distribution gradually became disordered and the resin matrix debris gradually increased. The resin debris at room temperature was mainly generated by the failure of the adhesive layer and fiber decoupling. Resin debris at high temperatures was produced by softening failure of the resin matrix and was mostly attached to the fiber periphery and interlayer bonds.

The analysis of the microscopic fracture morphology of the metal layer showed that after the tensile fracture damage of the metal layer occurred at ambient temperature, the fracture was relatively neat, and the fracture surface mainly consisted of nodular surfaces with obvious laminar fracture characteristics. In addition, the morphology mainly consisted of river lines, with a very small number of tough nests and decoupling steps, showing a quasi-decoupling fracture mode, which indicated a typical mixed ductile and brittle fracture mode at room temperature. And the plasticity performance was poor. With increasing temperature, the fracture of the magnesium alloy layer showed an uneven fracture surface and the degree of undulation of the fracture surface became larger and larger, showing a honeycomb shape. An obvious necking phenomenon appeared at both ends of the magnesium alloy layer, as well as a large number of tough nests and tearing ribs. In addition, the depth of tough nests increased, and relatively thin tearing ribs existed around the tough nests, showing the typical characteristics of ductile fracture. In the FML tensile test, there was a large amount of dislocation movement in the metal layer, which led to the dislocation accumulation and the accumulation of a large number of micro-pores. And with the increase in deformation, the walls of the micro-pores continued thinning and finally began gradually tearing, forming a large number of tough nests and increasing plastic deformation. Compared with low temperatures, the magnesium alloy layer at high temperatures presented better plastic deformation performance. The fracture deterioration mode of the magnesium alloy layer changed from the typical mixed ductile and brittle fracture mode to a combined mode dominated by plastic fracture. In summary, it can be analyzed that the damage failure modes of FMLs were a mixture of multiple damages.

**Figure 9:**
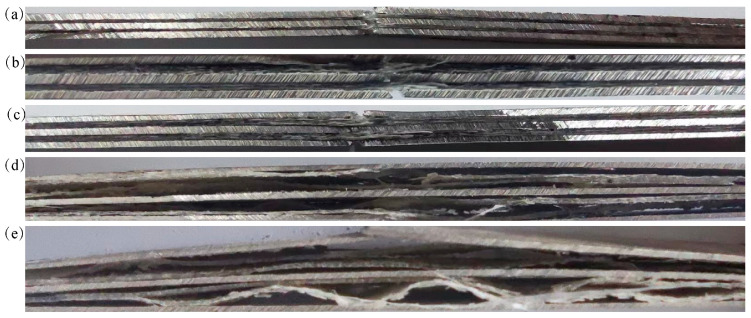
FML specimens with single-hole-specimen side surface after tensile test. (**a**) 25 °C, (**b**) 90 °C, (**c**) 120 °C, (**d**) 150 °C, (**e**) 180 °C.

Subsequently, Figure 9a clearly illustrates the presence of significant delamination between the magnesium alloy layer and the fiber layer following damage. During the progressive failure of the fiber metal laminate, due to variations in strain gradients between the magnesium alloy plies and fiber plies, damage occurred earlier in the fiber and matrix, thus leading to the delamination of magnesium alloy plies and fiber plies, and contributing to the loss of specimen integrity, the rapid expansion of ductile damage along the crack direction, and the reduction in the load-bearing weight capacity. The fiber layer carried most of the tensile load, and the fiber layer broke before the magnesium alloy layer, resulting in a precipitous drop in final load-carrying capacity. Delamination made the specimens lose its integrity, which was one of the reasons for the loss of bearing capacity of the specimens.

As depicted in Figure 9, when the temperature was below 120 °C, the fracture edges of the specimens were relatively flat, with slight delamination between the layers. When the temperature rose above 120 °C, the delamination damage became more severe, extending from the fracture location to both ends, and the decoupling of the resin and matrix in the fiber layer could be seen from the level of the fractured specimen. With the gradual increase in temperature and tensile load, the cohesive layer between fiber and metal layers showed an obvious damage phenomenon. In addition, it can be deduced that the softening of the matrix became more obvious when the ambient temperature exceeded the glass melting temperature of the matrix, and a significant presence of resin matrix fragments appeared between the layers. However, when the experimental temperature reached 180 °C, the transverse morphology demonstrated that the fiber layer did not show a more obvious fracture, and only the metal layer showed the first obvious fracture. After exceeding the limited working temperature of the epoxy resin matrix of 170 °C, the magnesium layers played the main role of bearing, and the fiber layers carried less. The bonding ability between the layers of FML played an important role in the tensile mechanical properties under different-temperature working conditions. Therefore, the higher the temperature, the more severe the delamination of the FML specimens and the lower the tensile residual strength. The morphology of FMLs under various temperatures showed that the fracture morphology below 90 °C was dominated by fiber fracture, and the damage of the fiber-matrix bond layer was dominated by debonding when the temperature exceeded 120 °C.

### 5.2. Tensile Mechanical Response of FMLs

**Figure 10:**
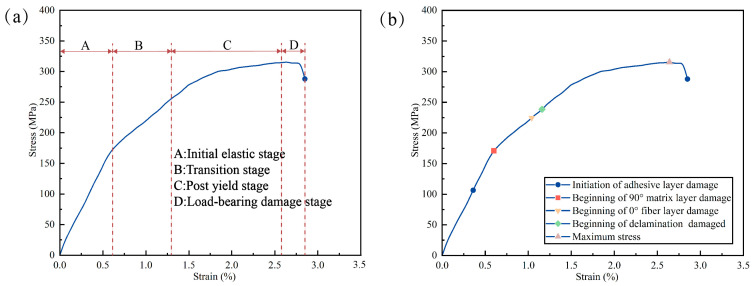
FML specimens with different stages of stress–strain curves. (**a**) Four stages, (**b**) failure sequences (single hole, 25 °C).

According to the methodology described in Section 2.2, multiple specimens were tested at each temperature range. Specifically, this paper covers tensile testing from the temperature range of 25–180 °C, and the data were recorded using the data acquisition system. The curves of the specimens under various temperatures had excellent similarity, which can ensure the reliability of experimental data. The typical stress–strain in Figure 10a can be divided into four phases: the initial elasticity stage, the over-yielding stage, the post-yielding stage, and the load-bearing damage stage. As shown in Figure 10b, the damage failure sequence of FML specimens was, firstly, failure fracture of the interlayer bonding layer, followed by damage to the 90° resin matrix layer, and then fiber breakage failure of the 0° fibers, followed by delamination damage and finally fracture. He and Chen et al.’s [37,38] research showed that a similar four-stage failure sequences conclusion was made in a study of tensile failure of fiber-reinforced aluminum alloy laminates.

It was more obvious that the tensile stress–strain curve was smoother when the temperature was below 90 °C. However, at temperatures higher than 120 °C, the stress–strain curve showed significant fluctuations as the temperature below 90 °C had less influence on the interlayer bonding layer and the resin matrix, along with a crisp ringing sound in the experiment. Therefore, the main damage mode of FML specimens below 90 °C was 0° fiber fracture with local fiber pull-out. Conversely, a different phenomenon occurred when the tensile ambient temperature exceeded 120 °C. Then, as the FML curve approached the ultimate load, the curve exhibited a sharp drop, the tensile load remained at a more stable level as the tensile displacement increased, and finally, the curve experienced a sudden drop until the specimen was completely fractured. This was because the epoxy resin matrix was subject to softening and failure as the temperature rose for the external temperature. When the temperature during stretching reached above 150 °C, the FML specimens showed a lot of matrix failure and softening, indicating that the resin was not suitable for working in an environment above 150 °C, and the specimens would inevitably show a lot of delamination.

**Figure 11:**
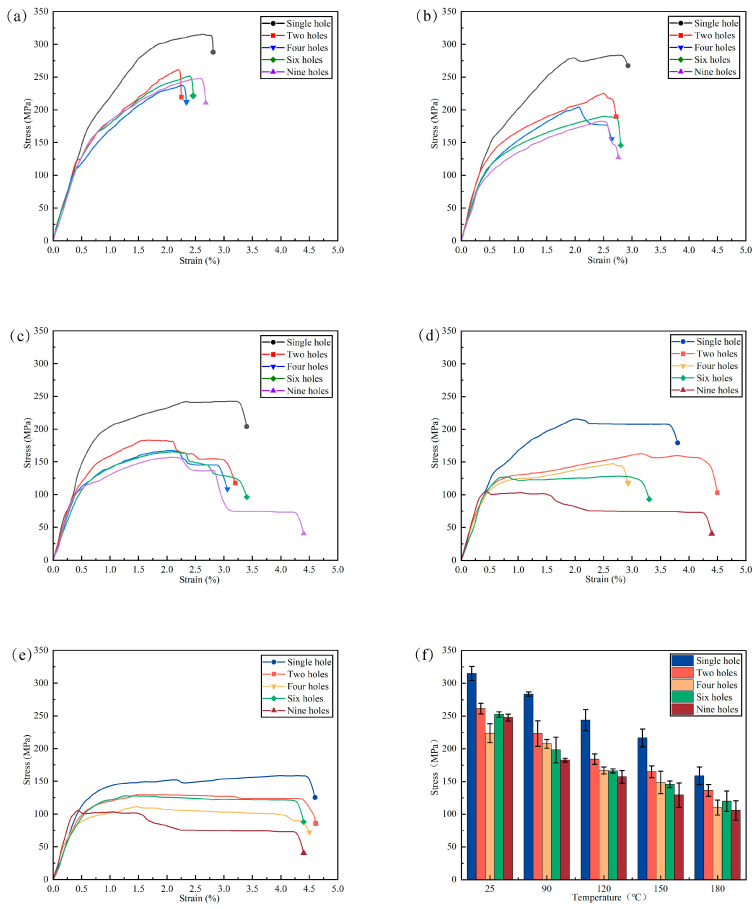
Mechanical responses and stress–strain curves of FML residual strength with different holes. (**a**) 25 °C, (**b**) 90 °C, (**c**) 120 °C, (**d**) 150 °C, (**e**) 180 °C, (**f**) residual stress.

As shown in Figure 11, the strength of the multi-hole specimens was significantly lower compared to the single-hole specimen, which was mainly due to the reduction in the load-bearing cross-sectional area of the specimens. However, from the comparison of stress–strain curves of 2-hole, 4-hole, and 6-hole specimens under various temperature environments, it could be concluded that the difference in tensile strength was small. Therefore, the number of holes had a smaller effect on the remaining strength in the same area of the bearing area, when the testing temperature of the specimens went from 25 °C to 180 °C, and the tensile strength of the specimens decreased by an average of 51.59%. As the number of holes increased, the critical damage range at the hole edges of the laminate expanded, resulting in stress redistribution and a decrease in the residual strength of the laminate. When the applied external load was close to the limit value, severe delamination could be noticed extending along the interface between the metal and fiber layers, and subsequently the specimen lost its load-bearing capacity. The proposed research by Yao et al. [39] on carbon-fiber-reinforced magnesium alloy laminate specimens subjected to tensile tests at different temperatures also found that delamination between laminates was more severe at higher temperatures.

**Figure 12:**
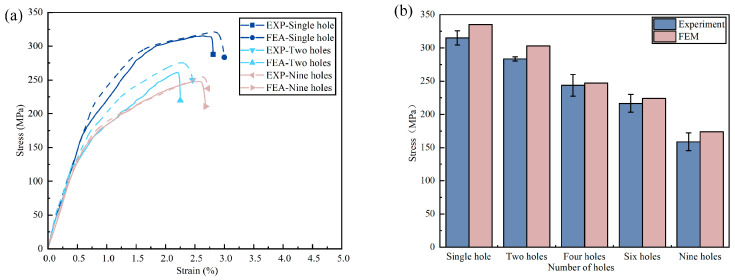
Comparison of finite element analysis and experimental residual strength of FMLs, (**a**) stress-strain curve comparison, (**b**) comparison of residual strength of specimens (25 °C).

Regarding Figure 12a, the curves from the experiments at room temperature and the predicted curves from the numerical simulations are in good agreement, with good agreement in the initial elastic phase and slight differences in the post-yield phase. In addition, a comparison of the finite element and experimental residual strengths of FMLs is shown in Figure 12b. The differences between the experimental and FEM results for the remaining strength are 6.09%, 6.93%, 1.82%, 3.62%, and 9.36%, respectively, with an average difference of 5.57%. Therefore, the numerical simulation results can match well with the experimental data, which is a reference value in the structural prediction of the strength of FMLs. Finally, due to certain structural defects in the machining process, the FEM prediction model is an ideal structure, so the FEM prediction results are always greater than the experimental measurement results, which is consistent with other studies.

## 6. Conclusions

In this paper, the damage evolution and failure mechanism of porous-fiber-reinforced magnesium alloy laminates under different temperature and hole number conditions were investigated by experimental and finite element methods, which can promote the application range of glass-fiber-reinforced magnesium alloy laminates. The VUMAT subroutine was integrated into ABAQUS/Explicit for numerical simulation analysis. The following conclusions were drawn.

The tensile load of glass-fiber-reinforced magnesium alloy laminate specimens decreased with increasing temperature, which showed a relatively obvious nonlinearity. When the working temperature exceeded 120 °C, the fracture strain of the specimen increased, and the residual strength decreased and the interface delamination damage was more obvious due to matrix softening and delamination damage failure. Therefore, the working temperature of FMLs is not recommended to exceed 120 °C.

The numerical simulations of the glass-fiber-reinforced magnesium alloy laminates were compared and the experimental mechanical response and damage failure patterns were in agreement. In addition, the average difference in the remaining strength values of the specimens at ambient temperature was 5.57%, and stress–strain curves were in good agreement. The delamination area of the specimens increased with the number of holes, and the delamination damage zone extended along the hollow edges in a funnel-like pattern to both sides. In terms of the mechanical response, the tensile strength of the specimens decreased by an average of 51.59%, since the residual tensile strengths of the porous FMLs (2–6 holes) were closer for the same cross-sectional area, and the nine-hole specimen had the lowest strength inside the specimens.

Regarding the sequence of damage of the specimens, it was relatively similar in terms of the variation in the number of holes at ambient temperature. The damage occurred first in the interlayer bond layer, followed by the damage of the 90° matrix layer, then the fracture of the 0° fiber layer and the fiber pull-out damage, and lastly, the fracture of the magnesium alloy layer due to plastic deformation. According to the finite element and experimental results, the damaged area of the interlayer bond layer increased with the number of holes.

At higher temperatures, the damage modes of glass-fiber-reinforced magnesium alloy laminates were significantly different from that existing at ambient temperature. The specimens mainly exhibited fiber fracture (pull-out) and resin matrix damage of the cohesive layer at ambient temperature, and matrix softening damage and interface delamination at higher temperatures. In contrast, the fracture of the magnesium alloy layer was mainly reflected by the transition from tough-brittle fracture to plastic fracture, which was manifested by the increase in elongation of the glass-fiber-reinforced magnesium alloy laminates specimens.

With the help of the SEM technique, the failure and different fracture damage models between the layers of glass-fiber-reinforced magnesium alloy laminates can be better observed, as well as the plastic cumulative damage modes of the magnesium alloy layers, which explains the decrease in the tensile properties of the specimens at different temperatures.

## Figures and Tables

**Table 1:** Mechanical properties of GFRP layer.

Property	Value	Property	Value
$E_{11}\;(\mathrm{GPa})$	49.65	$S_{23}\;(\mathrm{MPa})$	37
$E_{22}\;(\mathrm{GPa})$	12.9	$X_{T}\;(\mathrm{MPa})$	301
$E_{33}\;(\mathrm{GPa})$	12.9	$Y_{T}=Z_{T}\;(\mathrm{MPa})$	25
$G_{12}=G_{13}\;(\mathrm{GPa})$	5.33	$Y_{T}=Z_{T}\;(\mathrm{MPa})$	96
$G_{23}\;(\mathrm{GPa})$	5.33	$v_{12}=v_{13}$	0.34
$S_{12}=S_{13}\;(\mathrm{MPa})$	50	$v_{23}$	0.3

**Table 2:** Mechanical properties of AZ31B magnesium alloy sheet.

$\mathrm{Density}\;\rho \;(\mathrm{kg}/m^{3})$	Young’s Modulus E (GPa)	Yield Stress (GPa)	Poisson’s Ratio ν	Thickness t (mm)
1780	45	14	0.34	0.5

**Table 3:** Material parameters of the AZ31B magnesium alloy layer.

Johnson–Cook Constitutive Model Parameters				
*A* (MPa)	*B* (MPa)	*n*	*c*	*m*
172	360.73	0.45592	0.092	0.95
Fracture model parameters				
*d*_1_	*d*_2_	*d*_3_	*d*_4_	*d*_5_
−0.35	0.6025	−0.4537	0.206	7.2

**Table 4:** Material parameters of the cohesive layer elements.

*E* (GPa)			*t*° (MPa)			*G*_c_ (N/mm)			Density (kg/m^3^)	Ultimate Temperature (°C)
$E_{\mathrm{mm}}$	$E_{\mathrm{ss}}$	$E_{\mathrm{tt}}$	$\sigma_{n}^{0}$	$\sigma_{s}^{0}$	$\sigma_{t}^{0}$	$G_{IC}$	$G_{IIC}$	$G_{IIIC}$	$\rho_{c}$	T
2	0.75	0.75	65	38	38	2	4	4	920	180

## Data Availability

Not applicable.
